# Editorial: Training load in sport: current challenges and future perspectives, volume II

**DOI:** 10.3389/fspor.2026.1813915

**Published:** 2026-03-16

**Authors:** Luís Branquinho, Elias de França, Pedro Forte, José E. Teixeira, Ricardo Ferraz, Ronaldo Vagner Thomatieli-Santos

**Affiliations:** 1 Bioscience Superior School of Elvas, Polytechnic Institute of Portalegre, Elvas, Portugal; 2 Life Quality Research Center (LQRC-CIEQV), Santarem, Portugal; 3 Research Center in Sports Sciences, Health Sciences and Human Development, Covilhã, Portugal; 4 Centro de Investigação do Instituto Superior de Ciência Educativas (CI-ISCE), Penafiel, Portugal; 5 Interdisciplinar Graduate Program in Health Sciences, Universidade Federal de São Paulo, Santos, Brazil; 6 Human Movement Laboratory, São Judas University, São Paulo, Brazil; 7 São Paulo Futebol Clube, São Paulo, Brazil; 8 Department of Sports Sciences, Polytechnic Institute of Bragança, Bragança, Portugal; 9 Research Center for Active Living and Well-Being (Livewell), Polytechnic Institute of Bragança, Bragança, Portugal; 10 Department of Sports, Higher Institute of Educational Sciences of the Douro, Penafiel, Portugal; 11 Department of Sport Sciences, Polytechnic Institute of Cávado e Ave, Guimarães, Portugal; 12 SPRINT—Sport Physical Activity and Health Research & Inovation Center, Rio Maior, Portugal; 13 Sports Sciences Department, University of Beira Interior, Covilhã, Portugal; 14 Graduate Program in Psychobiology, Universidade Federal de São Paulo, São Paulo, Brazil

**Keywords:** athlete, injury prevention, performance, sport, training load

## Introduction

Monitoring and manipulating training load remain a central concern in sports science. The 14 articles in Volume II of the *Training Load in Sport: Current Challenges and Future Perspectives* Research Topic cover a broad spectrum of sports and methodological approaches. They collectively underscore that training load is not a one-size-fits-all constructs; instead, it must be tailored to the athlete's sport, competitive level and physiological/psychological profile. This editorial synthesizes the key findings, reflects on emerging themes and outlines directions for future research.

## Systematic reviews and meta-analyses

A core theme of this volume concerns evidence-based prescriptions for different training methods. In a systematic review, Tian et al. analysed dose–response relationships compared to hard-surface training. They concluded that sand surfaces improve change-of-direction and horizontal jump performance, particularly when programmes last longer than six weeks with three sessions per week of less than 40 min; benefits were greater for trained individuals in change-of-direction and for untrained individuals in horizontal jump (Wang et al.).

Hariss et al. reviewed cluster training and found that manipulating work-to-rest ratios using clusters enhances explosive power in sports such as volleyball and soccer; however, programmes should be tailored to sport-specific energy demands and individual athlete characteristics (Zhao et al.).

A meta-analysis on personalized warm-up strategies by Li et al. concluded that high-proficiency athletes with strong squat strength benefit from high-intensity pre-activation with short/medium recovery intervals, whereas low-proficiency athletes should prioritise strength development before using such strategies; optimal rest intervals also differ by gender and region (Xu et al.).

Finally, a narrative review of training-intensity distribution (TID) for cyclic endurance sports argued that no single model (polarised, pyramidal or threshold) is universally superior and that hybrid or sequential approaches may better suit an athlete's development; the selection should be flexible and individualized (Sun et al.).

## Strength-training innovations

Strength development features prominently in several original studies.

One study was related to Velocity-based resistance training (VBT) in male collegiate boxers improved maximum strength similarly to traditional percentage-based training but produced superior gains in vertical/horizontal jumps and sprint performance, supporting VBT as a tool for neuromuscular adaptation (Han et al.).

Another study on relative strength and the hang power clean/high pull found that the hang high pull's optimal load (−65% 1 RM) is lower than that of the hang power clean (−80% 1 RM); athletes with greater relative strength should train with higher loads to maintain maximal power output (Xie et al.).

Researchers compared stepwise load reduction training (SLRT) with conventional medium-load resistance training in healthy males. SLRT elicited greater strength gains and higher lactate responses, enhancing hypertrophy and endurance. However, the generalisability to women and team-sport athletes remains unknown (Zeng et al.).

In German national rowers, load–velocity characteristics were associated with 2 000-m ergometer performance. Besides maximal strength, power at high movement velocities (30%–50% 1 RM) correlated strongly with rowing performance, suggesting that high-velocity power training could augment endurance performance (Jacobs and Schumann).

## Specialised conditioning and anaerobic load

Sprint interval climbing added to training for elite female +78 kg judo athletes increased anaerobic training load without causing muscular damage or reducing fitness. The absence of a control group limits causal inference, but the work offers a practical strategy for judo coaches (Huang et al.).

Individual differences in training load among international-level handball players were analysed across congested match schedules. Athletes exhibited significant variation in weekly load, and compensatory training should be individualized to manage fatigue and maintain performance (Büchel et al.).

In rugby union, contact load was associated not only with contact injuries but also with non-contact injuries, indicating that monitoring contact events is important for preventing overuse injuries (Iwasaki et al.).

The session-RPE method proved reliable for quantifying workload in Olympic curling; despite subjective biases, it remains a simple tool for tracking load across phases and should be selected based on training objectives (Wu and Li).

Microcycle structures were compared across two competitive seasons in elite female Portuguese soccer. Adding a fourth training session per week increased accumulated load and training-monotony ratios (TMr) for several variables; however, more total distance was paradoxically covered in the three-session microcycle, and relative load differences were small, underscoring the importance of contextualizing load metrics (Oliveira et al.).

A study in elite tennis compared playing styles and match outcomes. Aggressive baseliners performed more high-intensity decelerations and had higher player-load values during volleys, smashes and blocks, whereas linear running distances did not differentiate winners from losers. The authors recommend focusing on technical–tactical skills and monitoring training load/RPE for periodization (Tóth et al.).

## Toward a holistic perspective on training load

Collectively, these studies reveal that training load should be interpreted through a multidimensional lens. Systematic reviews call for flexible and individualized training-intensity distributions, warm-up routines and cluster-set structures. Original research emphasises that maximal strength is necessary but not sufficient; power at high movement velocities, load distribution within and across microcycles, and athlete-specific responses to novel training modes (e.g., sand surfaces, sprint interval climbing, stepwise load reduction) are all critical.

The research also highlights monitoring strategies. Session-RPE remains valuable for simple contexts, but training-monotony ratios, contact load and individual weekly variation provide deeper insight. Sport-specific analyses (curling, tennis, handball, rugby, rowing, judo, boxing and soccer) illustrate the necessity of tailoring metrics to technical demands and injury profiles.

## Future directions

Despite substantial progress, several gaps persist. Most studies involve short-term interventions or cross-sectional designs. Longitudinal studies that track athletes across seasons are needed to understand chronic adaptation and injury risk. Additionally, research should integrate physiological measures (e.g., heart-rate variability, hormonal markers) with load metrics and explore psychological variables (motivation, sleep, stress) to adopt a biopsychosocial approach. Under-represented populations (women in strength-training studies, youth and para-athletes) should be prioritised. Finally, emerging technologies such as wearable sensors and machine-learning analytics offer opportunities for real-time monitoring and individualized load prescription.

## Conclusion

Volume II of the *Training Load* Research Topic demonstrates that managing and understanding training load is an evolving science requiring nuanced interpretation. From evidence-based prescriptions for sand training and cluster sets to insights on contact load and session-RPE monitoring, the articles emphasise that no single metric or model can capture the complexity of athletic training. Coaches and practitioners must integrate multiple sources of data, adjust for individual characteristics and remain mindful of the broader physiological and psychosocial context. The future of training-load research lies in comprehensive, individualized and data-driven approaches that enhance performance while safeguarding athlete health.

